# Mouse repeated electroconvulsive seizure (ECS) does not reverse social stress effects but does induce behavioral and hippocampal changes relevant to electroconvulsive therapy (ECT) side-effects in the treatment of depression

**DOI:** 10.1371/journal.pone.0184603

**Published:** 2017-09-14

**Authors:** Erin M. van Buel, Hannes Sigrist, Erich Seifritz, Lianne Fikse, Fokko J. Bosker, Robert A. Schoevers, Hans C. Klein, Christopher R. Pryce, Ulrich LM Eisel

**Affiliations:** 1 Groningen Institute for Evolutionary Life Sciences (GELIFES), University of Groningen, Groningen, Netherlands; 2 University of Groningen, University Medical Centre Groningen, Dept of Nuclear Medicine & Molecular Imaging, Groningen, Netherlands; 3 Research School of Behavioural and Cognitive Neurosciences, University of Groningen, Groningen, Netherlands; 4 Preclinical Laboratory for Translational Research into Affective Disorders (PLaTRAD), Psychiatric Hospital, University of Zurich, Zurich, Switzerland; 5 University of Groningen, University Medical Centre Groningen, Dept of Psychiatry, Groningen, Netherlands; Bilkent University, TURKEY

## Abstract

Electroconvulsive therapy (ECT) is an effective treatment for depression, but can have negative side effects including amnesia. The mechanisms of action underlying both the antidepressant and side effects of ECT are not well understood. An equivalent manipulation that is conducted in experimental animals is electroconvulsive seizure (ECS). Rodent studies have provided valuable insights into potential mechanisms underlying the antidepressant and side effects of ECT. However, relatively few studies have investigated the effects of ECS in animal models with a depression-relevant manipulation such as chronic stress. In the present study, mice were first exposed to chronic social stress (CSS) or a control procedure for 15 days followed by ECS or a sham procedure for 10 days. Behavioral effects were investigated using an auditory fear conditioning (learning) and expression (memory) test and a treadmill-running fatigue test. Thereafter, immunohistochemistry was conducted on brain material using the microglial marker Iba-1 and the cholinergic fibre marker ChAT. CSS did not increase fear learning and memory in the present experimental design; in both the control and CSS mice ECS reduced fear learning and fear memory expression. CSS induced the expected fatigue-like effect in the treadmill-running test; ECS induced increased fatigue in CSS and control mice. In CSS and control mice ECS induced inflammation in hippocampus in terms of increased expression of Iba-1 in radiatum of CA1 and CA3. CSS and ECS both reduced acetylcholine function in hippocampus as indicated by decreased expression of ChAT in several hippocampal sub-regions. Therefore, CSS increased fatigue and reduced hippocampal ChAT activity and, rather than reversing these effects, a repeated ECS regimen resulted in impaired fear learning-memory, increased fatigue, increased hippocampal Iba-1 expression, and decreased hippocampal ChAT expression. As such, the current model does not provide insights into the mechanism of ECT antidepressant function but does provide evidence for pathophysiological mechanisms that might contribute to important ECT side-effects.

## 1. Introduction

Electroconvulsive therapy (ECT) is one of the most effective therapies for major depressive disorder (MDD) [1]. One of the main indications for ECT is treatment-resistant depression, in which ECT reaches remission rates of around 50% [2]. In addition, due to its quick onset of clinical improvement, ECT is applied for the prevention of suicide in severe depression [3]. ECT involves the induction of brain seizures via electrodes that are placed on the patient’s scalp. ECT is typically given 2–3 times per week and clinical improvement is monitored closely. The total number of treatments that are required to achieve full remission of depressive symptoms varies between patients, with most patients achieving remission within four weeks after treatment onset [4]. Whilst ECT has only a few adverse effects, cognitive deficits, primarily in the form of anterograde and retrograde amnesia, are quite common and constitute a limitation on the clinical applicability of ECT [5]. These cognitive ECT side effects are typically transient, subsiding within weeks to months after discontinuation of treatment, with more severe and persistent cases of amnesia being relatively rare [5].

Despite its high efficacy as a depression treatment, the mechanisms by which ECT exerts its beneficial effects remain poorly understood. Animal studies investigating the effects of electroconvulsive seizures (ECS), the animal counterpart of ECT, offer unique opportunities to identify the mechanisms underlying the clinical effects and side effects of ECT. ECS experiments are typically conducted in rat or mouse and take the form of inducing seizures by applying electric current via ear clip electrodes. Studies differ in the number and frequency of ECS sessions, with sessions usually being given daily or once per 2–3 days, and a total of 5–10 sessions [6–8]. This variation is indicative of a lack of consensus on what might be an optimal ECS protocol in rodents.

To-date, the focus of rodent ECS experiments has been to study its impact on behavior and brain in otherwise non-manipulated, “healthy” rodents. With regards to behavior, it has been demonstrated, for example, that ECS induces increased mobility in the forced swim test, the same effect as acute antidepressant administration [9–11]. With regards to the brain, notable and consistent effects of ECS in rodents are increases in hippocampal neurogenesis [12,13] and in the expression of neurotrophic factors including brain derived neurotrophic factor (BDNF) and vascular endothelial growth factor (VEGF) [14–18]. These effects may indicate rearrangement of the cellular connectivity between hippocampus and its afferent and efferent projection areas [19,20]. In humans, ECT-induced neurogenesis and neurotrophic effects can be inferred indirectly from the observed increases in hippocampal volume and in serum/plasma levels of neurotrophic factors including BDNF and VEGF [14,21–25].

In order to increase the back-translational relevance of animal studies of ECS effects, it needs to be applied in animal models of depression-relevant pathology. A small number of such studies have been conducted, including the reversal by ECS of changes induced by chronic unpredictable mild stress or repeated corticosterone administration, or of depression-relevant traits induced by selected breeding e.g. Flinders Sensitive Line rat and Wistar-Kyoto rat. For each of these models, ECS has been demonstrated to normalize behavior in one or more behavioral tests, including the forced swim test, the sucrose preference test, and the open field test [26–30].

These studies have provided valuable insights into the mechanisms underlying the antidepressant and side effects of ECT. For example, ECS-induced reductions in depression-like behavior were found to be accompanied by normalization of BDNF and Neuropeptide Y expression in brain areas relevant to depression [27–30]. Other studies have focused on the mechanisms involved in ECS-induced memory impairment, with results pointing towards increased HPA-axis activity and decreased long-term potentiation as underlying factors [27,29]. Interestingly, memory-related side effects could be prevented by pre-treatment with propofol without affecting antidepressant effects, provided that ECS stimulus intensity was increased in propofol-treated rats, demonstrating the importance of performing ECS studies under clinically relevant conditions [29].

In mice, environmental manipulations based on the resident-intruder paradigm have been demonstrated to lead to depression-relevant changes in brain, physiology and behavior. In chronic social stress (CSS), C57BL/6 mice are exposed continually to mice of a dominant, aggressive strain, CD-1, including brief daily attacks, for 15 days. Relative to control mice, CSS mice exhibit altered brain functional connectivity [31], increased immune-inflammation in periphery and brain in terms of pro-inflammatory cytokines and the kynurenine pathway [32,33], decreased motivation for reward [34], increased Pavlovian fear learning-memory [32,33], increased learned helplessness [32], and increased physical fatigue [32]. It has been demonstrated that some CSS-induced reward deficits are attenuated by the antidepressant agomelatine [34], and that CSS-induced excessive fear memory is reversed by the antidepressant escitalopram as well as by an inhibitor of the kynurenine pathway [33]. That is, CSS induces changes in behavioral processes that are recognised as important dimensions in negative valence and positive valence domains in the research domain criteria (RDoC) framework for mental disorders [35]. These CSS effects are obtained without dividing mice into susceptible versus unsusceptible sub-groups based on their subsequent passive avoidance of the aggressor mouse strain, the method used with a 10-day chronic social defeat protocol [36]. That is, CSS has been applied using an inclusive experimental design, as used extensively by other groups with other stressors e.g. chronic unpredictable mild stress [37–39].

The principal aims of the current mouse study were to investigate the effects of ECS in terms of reversing the depression-relevant behavioral changes induced by CSS and to establish the concomitant changes in the hippocampus in terms of markers of microglia and acetylcholine function. The behavioral states of interest were, increases in fear learning-memory and fatigue. Such a model would be valuable for the back-translational study of the neurobiology underlying antidepressant effects and side effects of ECT.

Mice underwent CSS or control exposure, followed by 10 daily ECS or sham-control sessions, and were then tested in terms of fear learning-memory and physical fatigue. Whilst the expected CSS effect of increased fear learning-memory was not maintained in combination with the ECS/sham procedure, the expected CSS effect of increased fatigue was observed but was further increased, rather than being reversed, by ECS. Interestingly, however, repeated ECS resulted in impaired learning-memory, which is of relevance to the cognitive side effects that are a common feature of ECT. There is evidence that ECT/ECS-induced cognitive impairment is associated with structural and functional alterations in the cholinergic system [40,41]. There is also evidence that immune activation, including increased microglial activity, is increased after ECS [6,42], which may in turn induce short-term cognitive deficits [43]. Therefore, to investigate their involvement in the ECS-induced cognitive impairments observed in this study, we assessed hippocampal tissue for changes in immunohistochemical markers of these two systems: the microglial marker ionized calcium-binding adapter molecule (Iba-1) and the cholinergic marker choline acetyl transferase (ChAT). ECS increased microglial activity and decreased ChAT activity in the hippocampus of CSS and control mice, thus supporting a role for cholinergic alterations and neuroimmune activation in ECT/ECS-induced cognitive deficits.

## 2. Materials & methods

### 2.1. Animals

C57BL/6J male mice were bred in-house (Zürich), weaned at 3 weeks and housed in groups of 2–3 littermates up to the start of the experiment, at age 10 weeks. The CD-1 mice used for CSS were ex-breeder males aged 8 months obtained from Janvier (France), and single-housed up to the start of the experiment. Mice were kept in IVC cages on a reversed 12:12 light/dark cycle (lights off at 7 am), and at 20–22°C and 50–60% humidity. Food and water were available *ad libitum*. Mice were handled daily for 5 days before the start of the experiment. Measurement of body weight was performed daily for the entire duration of the experiment. As described below, mice were allocated to CSS or control by counterbalancing on their motor activity scores, followed by ECS or sham-procedure, yielding four treatment groups with n = 11–12 per group (CSSxSham and CSSxECS groups: n = 11, 11; CxSham and CxECS groups: n = 12, 12). The study was conducted under a permit (170/2012) issued by the Veterinary Office, Zürich, Switzerland.

### 2.2. Chronic social stress

Chronic social stress (CSS) was conducted as described in detail elsewhere [32]. Briefly, on the first day of CSS (study day 1), each CSS mouse was housed singly in the home cage of a CD-1 mouse separated by a transparent, perforated divider. The CSS mouse was then placed with the CD-1 mouse for either a cumulative total of 60 s physical attack or 10 min maximum. Each day for 15 days, between 12 and 4 pm, the CSS x CD-1 mouse pairings were rotated so that CSS mice were confronted daily with a novel CD-1 mouse; the CSS mouse remained in the compartment where the confrontation occurred while the CD-1 mouse was placed in the other compartment of its cage. To avoid bite wounds, the lower incisors of CD-1 mice were trimmed every third day across CSS. Control mice remained in littermate pairs and were handled and weighed daily.

### 2.3. Electroconvulsive seizures

Beginning on the day after 15-day CSS/control, half of the CSS and control mice received one daily electroconvulsive seizure (ECS) over a period of 10 days (study days 16–25). Between 3 and 5 pm, mice were anesthetized with isoflurane (3% in O_2_ at 800 ml/min) and the ears were cleaned with 70% alcohol. An electrical current of 80 mC (80 mA, 50 Hz, 1s duration and 0.5 ms pulse width) generated by an ECT unit (Ugo Basile, Italy) was applied via ear clip electrodes. This current induced a tonic-clonic seizure lasting 5–20 seconds, with an overall average seizure duration (± SD) of 13.0 ± 2.2 seconds (12.9 ± 2.2 for the control group and 13.1 ± 2.2 for the CSS group) Averages of individual mice over all 10 ECS sessions ranged from 11.1 to 14.5 seconds, which was comparable to seizure duration induced by ECS in other studies [42,44,45]. The other half of the mice underwent a sham procedure comprising anaesthesia, ear cleaning and electrode attachment in the absence of ECS.

### 2.4. Behavioral testing

#### 2.4.1. Motor activity test

Two motor activity tests were performed, the first prior to the onset of CSS (morning of study day 1) and the second on the day after the last CSS session (morning of study day 16). Using a Multi-Conditioning System (TSE Systems GmbH, Bad-Homburg, Germany [46]), the mouse was placed in an arena containing a grid floor. The distance the mouse moved and the percentage of time spent freezing were recorded via an infrared beam movement detection system. The first test provided baseline activity scores that were used to counterbalance allocation of mice to CSS and control groups [32]. The second test was used to assess CSS effects on motor activity, in particular whether there was any evidence for psychomotor retardation.

#### 2.4.2. Tone-shock fear learning and memory

Increased responsiveness to acute threat, including increased fear conditioning, is a common dimension in depression and other stress-related psychiatric disorders [35,47], and CSS increases tone-shock fear learning and memory [32,33]. Fear conditioning and expression testing [33,48] were conducted on two consecutive days, starting the day after the last ECS session (study day 26). For fear conditioning, the mouse was placed in the same arena as was used for the motor activity tests and exposed to six trials of a discrete, neutral tone of 5 kHz at 85 dB (conditioned stimulus, CS) presented via a loudspeaker for 20 sec. The final 2 sec were contiguous with a 2 sec x 0.15 mA inescapable foot shock (unconditioned stimulus, US) from the electrified grid floor. The inter-trial interval (ITI) was 120 s. For analysis, trials were grouped in CS blocks 1–2, 3–4, 5–6 and ITIs 1, 2–3, 4–5. The following day, for fear expression (memory) testing, the mouse was placed in the same arena and exposed to nine trials of the tone CS for 30 sec and with an ITI of 90 sec, in the absence of foot shocks. For analysis, trials were grouped into CS blocks 1–3, 4–6, 7–9, and ITIs 1–2, 3–5, 6–8. During each session, percentage of time spent freezing was recorded during the CS and ITI.

#### 2.4.3. Treadmill fatigue test

The treadmill fatigue test requires the mouse to run on an inclined treadmill to avoid or escape an electrified floor at the base of the treadmill [32,49]. Fatigue is a common symptom in depression (DSM-5, ICD-10). Reduced treadmill running is induced by CSS [32] and by depletion of dopamine in the nucleus accumbens [34]. In contrast to the forced swim test and tail suspension test, where it is unclear whether passivity indicates an adaptive, energy-conserving strategy or a cessation of active coping [50], in the treadmill fatigue test it is clear that passivity is maladaptive and related to fatigue. On two days after the fear expression test (study days 28–29), mice were studied in a treadmill fatigue test [32], conducted using a mouse single lane treadmill (Panlab/Harvard Apparatus, Cornellà, Spain) inclined at 5° with an electrified grid (0.15 mA) at its lower end, which mice could avoid or escape by running uphill. On the first day, the habituation session consisted of 2 min at a treadmill speed of 0 cm/sec, 5 min at 15–20 cm/sec at 1 min increments, and 5 min at 20 cm/sec. The following day, first a warm-up test was performed, consisting of 2 min at 0 cm/sec and 5 min at 20 cm/sec. Immediately afterwards, a test session was conducted at a treadmill speed of 23 cm/sec. The total number and duration of shocks the mouse received were scored automatically. For the test session the maximum duration was 20 min (1200 sec); if a mouse reached a total cumulative duration of 10 sec foot shock, the test was stopped immediately and the test duration up to this point was scored.

#### 2.4.4. Hot plate test

In a subset of mice (n = 6 per group) a hot plate test [32,46] was conducted immediately after the treadmill fatigue test, in order to assess whether group differences were related to differences in nociception. The mouse was placed on a plate with a temperature of 50°C. The latency (sec) until the display of one of several pain-related behaviors (licking a forepaw, licking a hind paw, lifting a hind paw or jumping) was scored, after which the mouse was immediately removed from the hot plate and replaced in its home cage. The maximum duration of the test was 60 seconds.

### 2.5. Immunohistochemistry

At 1 day after completion of behavioral testing (study day 30), mice were deeply anesthetized (Pentobarbital) and perfused transcardially with 0.1M phosphate buffered saline (PBS) followed by a 4% paraformaldehyde (PFA) solution in 0.1M phosphate buffer. The brain was postfixed for 24 hours in 4% PFA in 0.1M PBS, followed by 18 hours in 0.1M PBS containing 30% sucrose. Brains were snap frozen on dry ice, temporarily stored at -80°C, and cut into sections of 20 μm.

#### 2.5.1. Ionized calcium-binding adaptor molecule 1 (Iba-1)

The microglial marker Iba-1 was visualized by a 3,3'-diaminobenzidine (DAB) staining. Coronal sections were selected for the following areas: prefrontal cortex (PFC), dorsal hippocampus, and ventral tegmental area (VTA). Sections were incubated for 72 hours with a 1:2500 dilution of primary antibody (rabbit anti-Iba1, Wako Chemicals, Neuss, Germany) in 0.01M PBS (pH 7.4) containing 1% bovine serum albumin (BSA) and 0.1% tritonX-100, followed by a 2-hour incubation with a 1:500 dilution of secondary antibody (goat anti-rabbit, Jackson ImmunoResearch, Suffolk, UK) in 0.01M PBS. Subsequently, sections were incubated for 1 hour with avidin-biotin complex (1:500; Vector Laboratories Burlingame, CA, USA) in 0.01M PBS. Finally, 0.075 mg/mL DAB was added and the DAB reaction was initiated with 100 μl 0.1% H_2_O_2_. After each step of the protocol, sections were rinsed with 0.01M PBS.

Analysis of microglial activation was performed with Image Pro software (Image-Pro Plus 6.0.0.26, Media Cybernetics Inc., Rockville, USA) using a method described by Hovens *et al*. [51]. Briefly, the software was used to determine the percentage of area covered by microglia cells (“total microglial cell size”) and the percentage of area covered by microglial cell bodies (“total size of the microglial cell bodies”). The ratio total size of the microglial cell bodies: total microglial cell size (cb/c) reflects activation state in such a way that higher values reflect increased microglial activity [51]. An investigator blinded to group assignment performed all analyses.

#### 2.5.2. Choline acetyltransferase (ChAT)

An immunohistochemical staining for choline acetyltransferase (ChAT) was performed to quantify cholinergic fiber density in hippocampal areas. Free-floating hippocampal sections were pre-incubated for one hour in 0.01M PBS containing 5% normal rabbit serum (NRS) and 0.4% Triton X-100 before incubation for 72 hours with a 1:333 dilution of goat anti-ChAT primary antibody (Merck Millipore, Amsterdam, Netherlands). This was followed by a 24-hour incubation with secondary antibody (rabbit anti-goat, Jackson ImmunoResearch, Suffolk, UK) in PBS containing 1% NRS, 0.5% BSA, and 0.2% Triton X-100. Subsequently, sections were incubated for 1 hour with avidin-biotin complex (1:500; Vector Laboratories Burlingame, CA, USA) in 0.01M PBS containing 0.2% TritonX-100. The DAB reaction was initiated by addition of 100 μl 0.1% H_2_O_2_ to a 0.075 mg/mL DAB solution containing 0.5 mg/ml ammonium nickel sulphate. After each step, sections were rinsed thoroughly with 0.01M PBS. Images were visualized using a microscope (Leica Microsystems, Rijswijk, Netherlands) at 400x magnification. Leica Application Suite (LAS) microscope software (Leica Microsystems, Rijswijk, Netherlands) was used to quantify the percentage of area covered by cholinergic fibers (fiber density). An investigator blinded to group assignment performed all analyses.

### 2.6. Statistical analysis

The basic statistical model used was 2 groups (CSS, Control) x 2 treatments (ECS, Sham). Statistical analysis was conducted using either GraphPad Prism 5.0 (GraphPad Software, San Diego, California, USA) or, in the case of 2- and 3-way repeated measure ANOVA, StatSoft Statistica 8.0. Significant interaction effects were analysed using a Bonferroni post hoc test. Statistical significance was set at p ≤ 0.05. All graphs were created in GraphPad Prism 5.0, using mean ± sem.

## 3. Results

### 3.1. Effects of CSS and ECS on body weight

**Fig 1:**
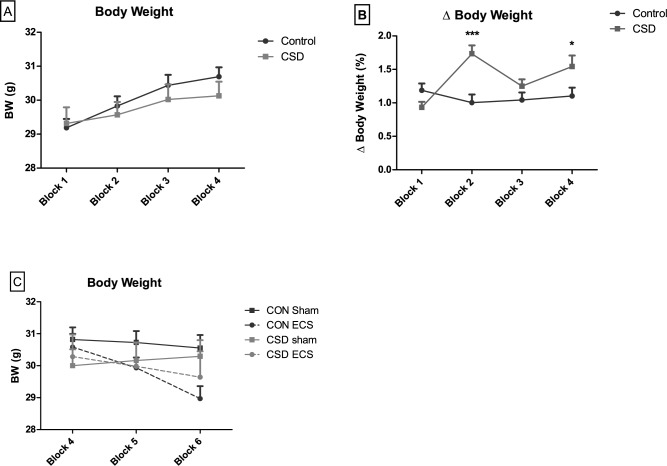
Comparison of body weight in CSS (n = 22) and control (n = 22) mice prior to and during ECS. A: Average body weight (g) in 5-day blocks prior to (block 1) and throughout the 15-day CSS procedure (block 2–4). B: Percentage average daily absolute (increase or decrease) body weight delta during the same period. (C): Average body weight during the final 5 days of CSS (block 4) and throughout the 10-day ECS procedure (block 5–6). Data presented as mean + sem. *p<0.05; ***p<0.001; two-way ANOVA with Bonferroni post hoc test.

The duration of daily CSS attack time was 52.1 ± 0.9 sec (mean ± sem), with a minimum of 30 sec and a maximum of 60 sec per session. A minority (n = 5) of 22 CSS mice were observed to fight back during 1–4 of the first 4 days of the protocol, and on days 5–15 all CSS mice displayed only submissive behavior during each session. Absolute body weight was measured daily and averaged over blocks of five days, with block 1 covering the period prior to CSS, and blocks 2, 3 and 4 covering the 15-day CSS protocol. There was a significant group X time interaction effect (p = 0.02), albeit in the absence of a group effect in any block (Fig 1A, S1 Table). For absolute day-to-day body weight variability (% ΔBW; % body weight change across two consecutive days, averaged over five days), there was a group x time interaction (p = 0.0001): absolute ΔBW was increased in CSS compared to control mice in block 2 and 4 (Fig 1B, S1 Table). Increased percentage daily absolute ΔBW, which reflects daily body weight change regardless of whether it is an increase or decrease, provides a reproducible marker for CSS efficacy [31–33].

To assess for group x treatment x time effects on average body weight, block 4 and two blocks covering the 10-day ECS protocol were analysed (Fig 1C, S1 Table). In the absence of a 3-way interaction effect, there was a group x time interaction (p = 0.003) and a treatment x time interaction (p<0.0001); however, post hoc tests did not reveal any block-specific significant effects of either group or treatment.

### 3.2. CSS without effect and ECS decreases fear learning and memory

**Fig 2:**
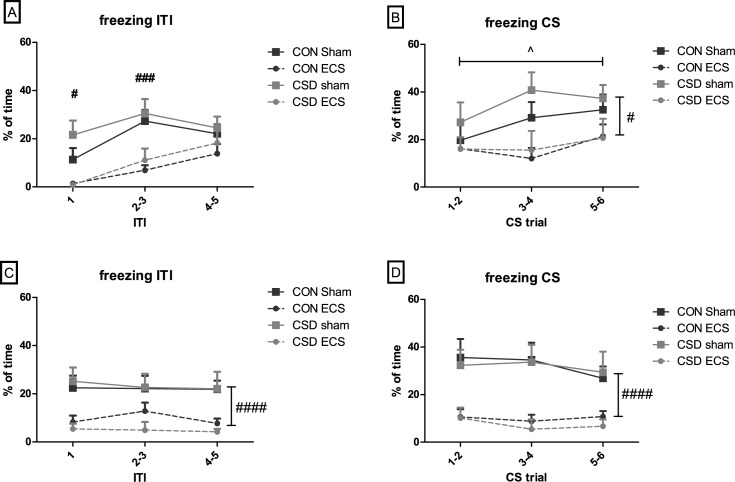
Effects of CSS and ECS on tone-shock (CS-US) fear conditioning and expression, measured as % time spent freezing. A + B: Fear conditioning stage; (A) Inter-trial intervals between CS-US pairings, (B) During CS of CS-US trials. C + D: Fear expression stage; (C) Inter-trial intervals between CS, (D) During CS trials. Data presented as mean + sem. In (A), significant treatment x trial interaction and significant trial-specific treatment effects in post hoc tests # p<0.05, ### p<0.001. In (B), significant main effect of trial ^ p<0.05, and significant main effect of treatment # p<0.05. In (C) and (D), significant main effect of treatment #### p<0.0001.

In the activity test conducted on study day 16, there was no difference in locomotor distance between CSS (78980±6932 arbitrary units (a.u.)) and control (83430±5944 a.u.) mice (p = 0.63). Following the 10-day ECS/sham treatments, tests of CS-US fear conditioning (study day 26) and CS fear expression (day 27) were conducted, with % time spent freezing used to quantify fear learning-memory. In contrast to previous CSS studies [32,33], there was no effect of CSS on fear conditioning (p = 0.26 and p = 0,41 for freezing during ITIs and CS respectively) or expression (p = 0,62 and p = 0,74 for freezing during ITIs and CS respectively). At fear conditioning, during the ITIs between CS-US pairings (Fig 2A, S2 Table) there was a treatment x trial interaction (p = 0.03); ECS mice exhibited less freezing than sham mice at ITI 1 and 2–3. During the CS of CS-US trials (Fig 2B, S2 Table) there was a main effect of trial (p = 0.03) with freezing increasing across trials, and a main effect of treatment (p = 0.01) with ECS mice exhibiting less conditioned freezing than sham mice. At fear expression, during ITIs between CS trials (Fig 2C, S2 Table) and during CS trials (Fig 2D, S2 Table) there was a main effect of treatment (p<0.0001 for ITI and CS) with ECS mice expressing less freezing than sham mice.

### 3.3. Both CSS and ECS decrease running in the treadmill fatigue test

**Fig 3:**
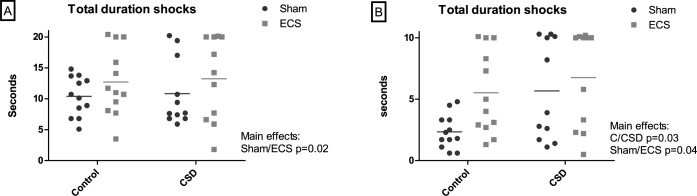
**Total duration of foot shock received in the treadmill fatigue test: (A) habituation phase, (B) test phase.** In (A), habituation comprised 2 min at a speed of 0 cm/sec, 5 min at 15–20 cm/sec at 1 min increments, and 5 min at 20 cm/sec. In (B), the maximum cumulative duration of foot shock allowed was 10 sec and then the test was terminated; otherwise the maximum test duration was 20 min.

Both CSS and ECS attenuated running in the treadmill fatigue test. At the habituation phase (study day 28, Fig 3A, S3 Table), there was no CSS effect, whilst the total duration of foot shock received was increased in ECS relative to sham mice (main effect of treatment, p<0.02). At the test phase (day 29 Fig 3B, S3 Table), there were main effects of group (p<0.03) and treatment (p<0.04) on total duration of foot shock received; CSS mice received more foot shock than control mice, and ECS mice received more foot shock than sham mice.

### 3.4. Pain sensitivity is not altered by CSS or ECS

**Fig 4:**
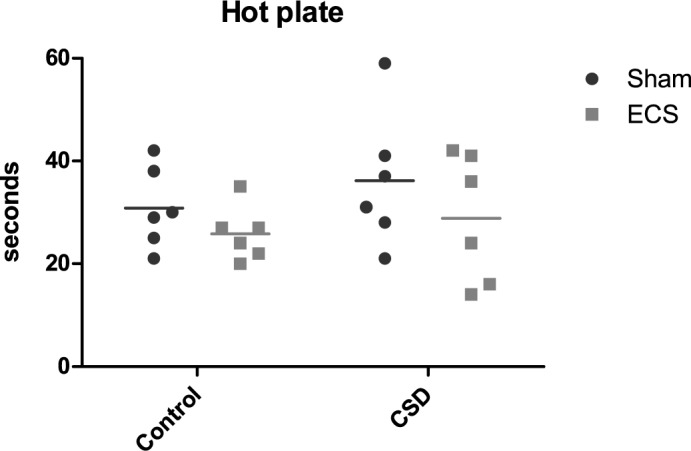
Hot plate test: latency to first display a pain-related behavior. There was no significant effect of CSS or ECS.

Pain sensitivity was measured in a subset of mice with the hot plate test (Fig 4, S4 Table). No significant effects were obtained for either CSS (p = 0.33) or ECS (p = 0.16).

### 3.5. CSS without effect and ECS increases microglial activity in hippocampal CA1 and CA3 regions

**Fig 5:**
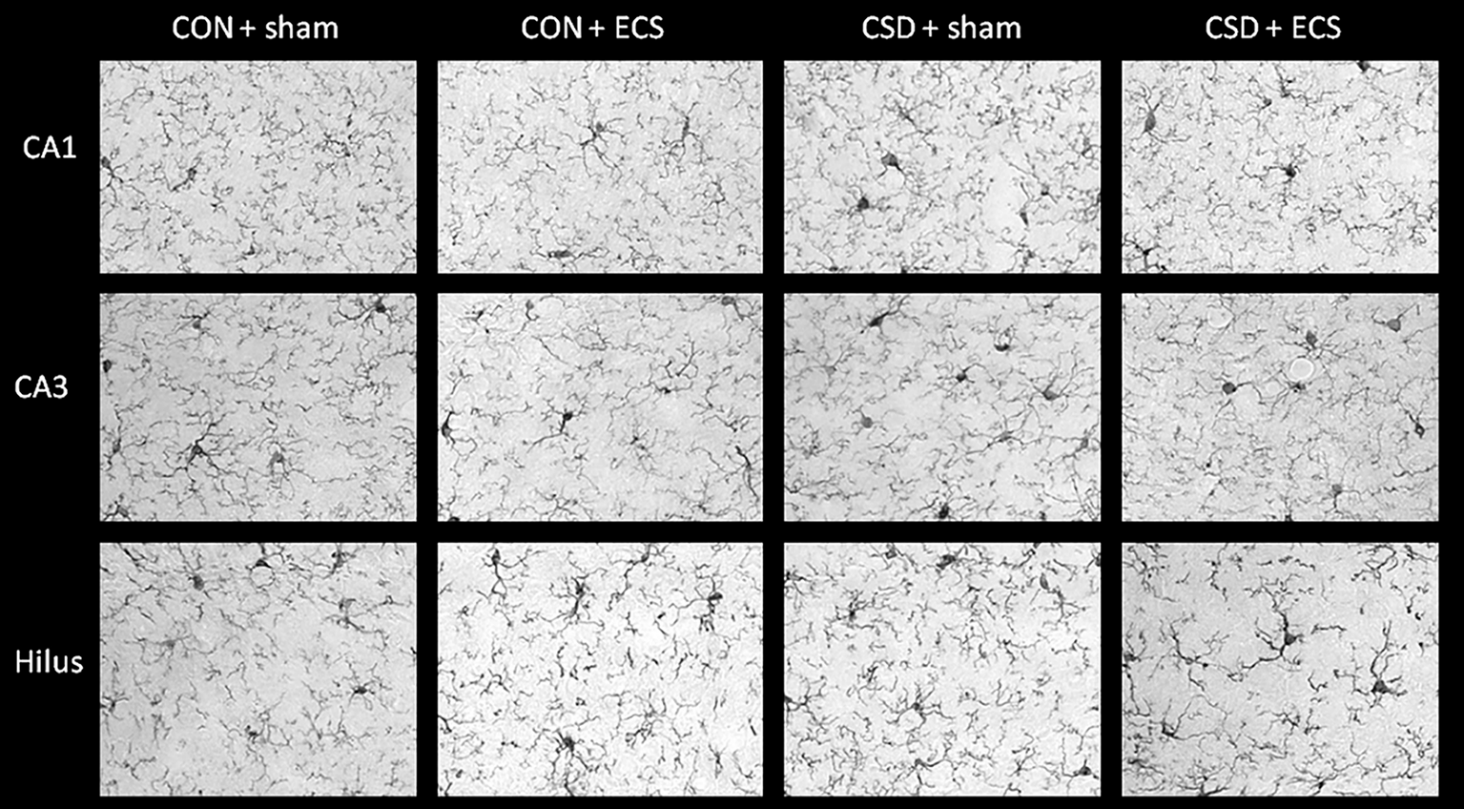
Iba-1 immunohistochemistry for the calculation of microglial activity: representative images of the hippocampal CA1 radiatum, CA3 radiatum and hippocampal hilus regions from control-sham, control ECS, CSS-sham, CSS-ECS mice.

An Iba-1 staining was conducted to measure microglial activity, which is reflected by the ratio between percentages of area covered by microglial cell bodies and by total microglial cell size (cb/c). The cb/c ratio was analysed for the hippocampal areas CA1 and CA3 oriens and radiatum, dentate gyrus molecular layer and hilus (Fig 5), and for the prefrontal cortex (PFC) and the ventral tegmental area (VTA). There were no significant CSS effects (Table 1). Mice that underwent ECS exhibited an increased cb/c ratio, indicative of increased microglial activity, for the radiatum of the hippocampal CA1 and CA3 regions specifically (Fig 5; Table 1).

**Table 1:** Microglia cell body:total coverage ratio based on Iba-1 immunohistochemistry.

	Mean ± sem				p-value		
	CON + sham	CSS + sham	CON + ECS	CSS + ECS	Interaction	Main effect	
						Group	Treatment
**CA1oriens**	0.016± 0.003	0.024± 0.004	0.021± 0.004	0.023± 0.003	0.38	0.18	0.47
**CA1radiatum**	0.018 ± 0.003	0.021± 0.001	0.026± 0.002	0.025± 0.002	0.35	0.65	0.01*
**CA3oriens**	0.021 ±± 0.002	0.023± 0.002	0.024± 0.002	0.020± 0.002	0.16	0.66	0.86
**CA3radiatum**	0.017 ± 0.002	0.019± 0.002	0.021± 0.001	0.024± 0.002	0.98	0.17	0.01*
**Dentategyrus**	0.025 ± 0.003	0.030± 0.003	0.032± 0.002	0.028± 0.002	0.96	0.32	0.28
**Hilus**	0.032 ± 0.005	0.036± 0.003	0.036± 0.003	0.040± 0.003	0.87	0.28	0.83
**PFC**	0.030 ± 0.005	0.033± 0.003	0.030± 0.003	0.034± 0.002	0.08	0.87	0.30
**VTA**	0.010 ± 0.002	0.009± 0.002	0.010± 0.002	0.009± 0.001	0.90	0.76	0.85

PFC = prefrontal cortex; VTA = ventral tegmental area.

Asterisks represent significance

*p<0.05.

### 3.6. Both CSS and ECS reduce hippocampal cholinergic fiber density

**Fig 6:**
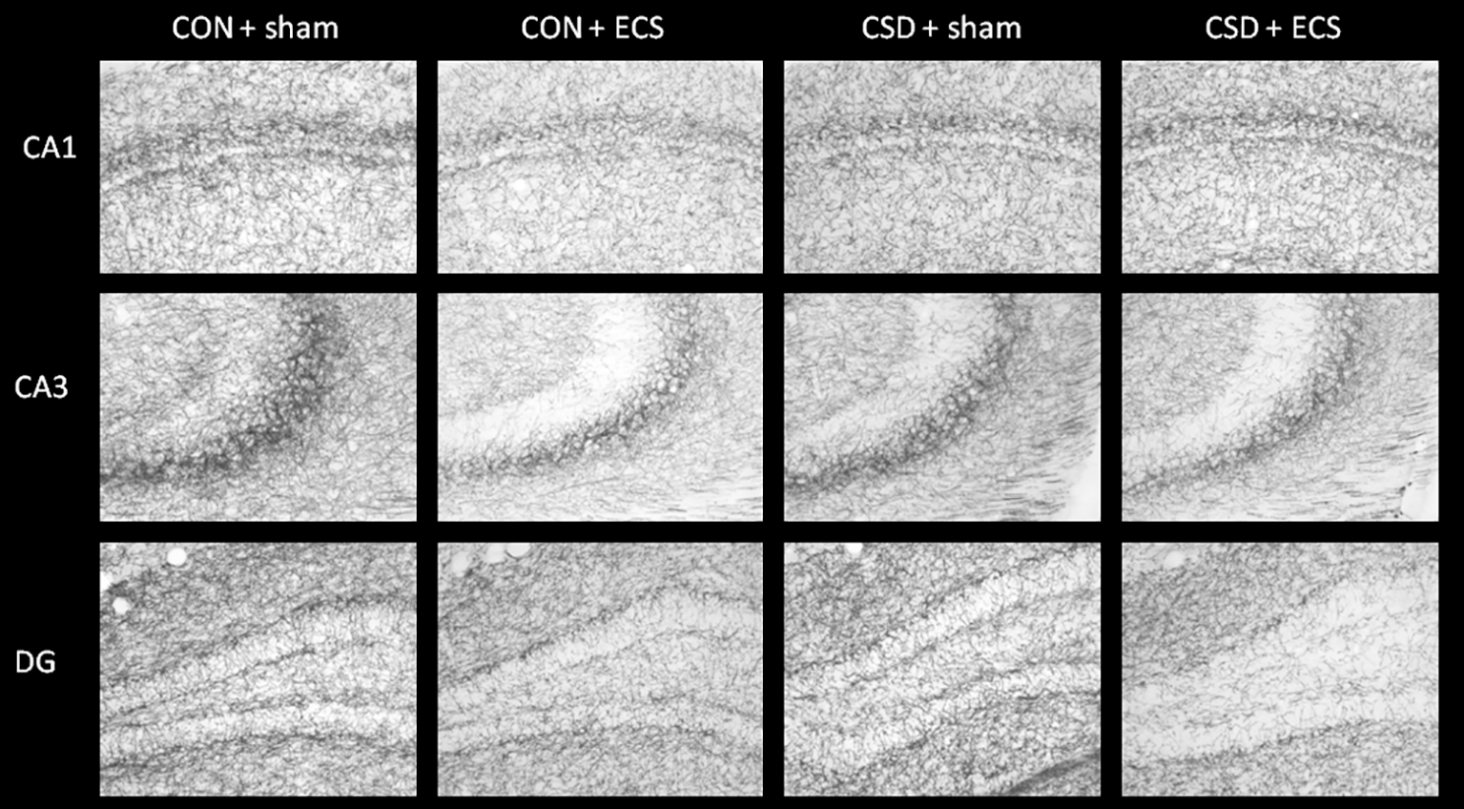
ChAT immunohistochemistry: representative images of the hippocampal CA1, CA3, and dentate gyrus (DG) regions from control-sham, control ECS, CSS-sham, CSS-ECS mice.

An immunohistochemical staining for choline acetyltransferase (ChAT) was conducted to quantify cholinergic fiber density in hippocampal areas (Fig 6; Table 2). For each of the CA1 pyramidal layer and CA3 pyramidal layer there was a significant group x treatment interaction effect (p = 0.03 and p = 0.02, respectively); CSS-sham and control-ECS mice each exhibited reduced cholinergic fiber density compared to control-sham mice in these regions. For each of the regions, CA3 oriens, stratum lucidens and radiatum, the molecular layer of the dentate gyrus and the hilus, there was a significant main effect of treatment, indicating reduced ChAT signal in ECS relative to sham mice (Table 2).

**Table 2:** Hippocampal cholinergic fibre density based on area covered by ChAT using immunohistochemistry.

	Mean ± sem				p-value		
	CON + sham	CSS + sham	CON + ECS	CSS + ECS	Interaction	Main effect	
						Group	Treatment
**CA1oriens**	30.31± 1.51	25.32± 2.47	25.40± 0.87	26.14± 1.05	0.07	0.17	0.19
**CA1 PL**	42.67± 1.50	36.10± 2.51	38.89± 1.24	40.65± 1.18	0.03*	0.41	0.57
**CA1radiatum**	30.14± 1.56	23.44± 2.21	25.49± 0.84	24.81± 1.24	0.06	0.22	0.10
**CA3oriens**	41.62± 2.06	33.29± 2.56	33.40± 0.77	33.92± 1.92	0.07	0.49	0.04*
**CA3 PL**	56.79± 1.82	49.19± 1.91	50.02± 1.00	52.30± 1.41	0.02*	0.45	0.06
**CA3 SL**	27.47± 2.23	19.89± 3.00	16.07± 1.47	18.78± 1.18	0.23	0.52	<0.001***
**CA3radiatum**	37.32± 2.30	30.28± 2.74	30.64± 1.03	30.18± 1.63	0.30	0.56	0.02*
**Dentategyrus GCL**	43.24± 1.44	39.23± 1.70	40.27± 1.13	39.82± 1.01	0.08	0.41	0.06
**Dentategyrus ML**	28.78± 1.65	25.28± 2.41	24.11± 0.94	23.99± 1.68	0.27	0.63	0.004**
**Hilus**	29.42± 1.61	25.29± 1.79	23.10± 1.11	22.79± 1.20	0.20	0.30	<0.001***

PL = pyramidal layer; SL = stratum lucidum; GCL = granule cell layer; ML = molecular layer.

Asterisks indicate significance

*p<0.05

**p<0.01

***p<0.001.

P-values are given for the interaction, main effect for treatment (Sham/ECS) and main effect for group (CON/CSS).

## 4. Discussion

In the present study design, the exposure of mice to CSS did not induce the expected increase in fear learning-memory but did induce the expected increase in physical fatigue. ECS impaired fear learning and memory in CSS and control mice and also increased physical fatigue in CSS and control mice. CSS was without effect on Iba-1 expression in the hippocampus, while ECS increased Iba-1 expression in hippocampus of CSS and control mice. CSS decreased hippocampal ChAT expression, and ECS also decreased hippocampal ChAT expression in CSS and control mice. Therefore, using a mouse model of CSS-induced reactivity to aversive challenge it was not possible to normalize behavior with ECS and thereby provide insights into the mechanism of ECT antidepressant function. However, the ECS protocol used resulted in behavioral effects of anterograde amnesia and fatigue, of direct relevance to the observed side effects of ECT in depressed patients, and the associated hippocampal effects of ECS are of potential relevance in this respect.

Chronic social stress mice exhibited increased daily body weight variability compared with controls during the 15-day procedure, providing positive biomarker evidence for CSS efficacy in this study [31–33]. The model of CSS-induced hyper-fear learning and memory was selected because of its robustness and reproducibility in previous studies (e.g. [35,36]), and because it would allow for the study of ECS impact in terms of therapeutic-like reversal of increased aversion reactivity but also side effect-like induction of learning-memory impairment. In the present study CSS was without effect on Pavlovian fear learning-memory. Compared with previous studies using the same test conditions (e.g. [33]), CSS-sham mice acquired a lower amount of freezing during conditioning and expressed less freezing on the following day, while control-sham mice exhibited freezing levels typical for this condition. In previous studies this test has been carried out 2–3 days after the 15-day CSS, and in the present study the interval between CSS and testing was 10 days, perhaps indicating that the CSS effect is limited in terms of its longevity. Another difference between the present study and typical study conditions was that mice underwent isoflurane anaesthesia on each of these intervening days; however, as noted above, control-sham mice exhibited expected levels of fear learning-memory. The model of CSS-induced attenuated physical effort on an electrified treadmill was also selected in relation to its demonstrated robustness [32] and because it would allow for the study of ECS therapeutic-like reversal induction of fatigue. CSS resulted in the expected deficit in treadmill running; testing was conducted 14 days after the end of CSS confirming the longevity of this CSS fatigue effect [35]. CSS was without effect in the hot plate test of pain-sensitivity, as reported previously [32].

The repeated ECS protocol used resulted in reduced learning of the Pavlovian association between the auditory CS and foot shock US, as demonstrated in both CSS and control mice. Furthermore, the subsequent expression of the association was, at the statistical level, even more attenuated by ECS, and again in CSS and control mice. These effects are consistent with ECS-induced impaired emotional learning and, in addition, impaired long-term (over-night) consolidation and/or recall. CS-US learning and memory are mediated primarily in the amygdala [52,53]. ECS-induced deficits in freezing were also observed in the intervals between successive CS presentations, both during acquisition and expression; in part these measures are likely to reflect learning and memory of the association of the general context with foot shock, and as such impaired function of the hippocampus is also implicated [54]. ECS, in addition to CSS, increased fatigue in the electrified treadmill test in CSS and CON mice. This ECS effect was even apparent during the habituation phase of the test, when a relatively low treadmill speed was applied to allow the mice to become acquainted with the treadmill and acquire the required operant escape-avoid responses. Therefore, it is possible that ECS impaired the operant active avoidance learning that is essential for adaptive behavior in the treadmill test. The CSS effect, and possibly to some extent also the ECS effect, is consistent with reduction in the level of effort mice were able/motivated to exert to avoid-escape foot shock [32]. A similar effect was induced by pharmacological depletion of dopamine in the nucleus accumbens [34]. CSS was without effect in the hot plate test of pain-sensitivity.

Therefore, the present data demonstrate that the repeated ECS protocol used induces anterograde learning and memory deficits. Several previous studies have reported ECS-induced learning and memory deficits in rodents [55–58]. Cognitive impairment, particularly anterograde and retrograde amnesia, is one of the most common side effects of ECT (for reviews, see [5,59]). The mechanisms underlying ECT-induced memory deficits are currently unknown, but structural and functional alterations in the cholinergic system have been proposed to be involved [40,41]. In line with this hypothesis, the current study provides evidence for cholinergic abnormalities after ECS in multiple hippocampal regions. The cholinergic system is essential for adaptive learning and memory, and thus the ECS-related cognitive deficits observed in this study may well be related to the co-occurring changes in ChAT-based cholinergic fiber density. Indeed, a recent study demonstrated that ECS-induced memory disturbances in rodents could be prevented by the cholinesterase inhibitor physostigmine and by the α4β2 nicotinic acetylcholine receptor (nAchR) agonist ABT-418, but not by the α7 nAchR agonist anabasine [41]. Further evidence for α4 nAchR involvement is provided by the finding that ECS reduces its expression in rodent prefrontal cortex and hippocampus [41]. With respect to human ECT, the cholinergic system and memory deficits, it has been reported that the acetylcholinesterase inhibitor rivastigmine enhanced memory in ECT-treated schizophrenia patients [40]. Together, these findings support the hypothesis that reduced synaptic signalling in the cholinergic system constitutes an important component of the ECS- and ECT-induced CNS changes underlying the major side-effect of cognitive impairment. Short and long-term rearrangement of connections from the hippocampus to frontotemporal connections and the amygdala are considered underlying mechanisms [19,20], which might also be the cause of severe memory problems that are sometimes observed during the course of ECT. The potential prevention of cognitive side effects by manipulations of the cholinergic system such as co-treatment with acetylcholinesterase inhibitors and acetylcholine receptor agonists in the CSSxECS paradigm applied in the current study would be an interesting direction for future research.

In addition to ECS effects in terms of reduced hippocampal ChAT expression, CSS led to reduced ChAT expression in the CA1 and CA3 pyramidal regions of sham mice specifically. These findings add to the existing evidence for abnormal central cholinergic functioning in chronic stress models, in the form of altered acetylcholinesterase levels and activity [60–65]. Indeed, it has been suggested that cholinergic deficits play an important role in stress-induced cognitive deficits [66].

Altered Iba-1 expression consistent with increased microglial activity was also observed in hippocampus of ECS mice, albeit in the radiatum of CA1 and CA3 regions, which did not exhibit cholinergic changes. There was no effect of ECS on Iba-1 expression in other hippocampal sub-regions, or in the prefrontal cortex or ventral tegmental area. Several previous studies have investigated ECS effects on microglial activity and findings are somewhat inconsistent [6,42,67–69]. Immune system activation is proposed to lead to temporary cognitive deficits including memory impairment, mediated by the hippocampus (for review, see [43]). Therefore, increased microglial activity might contribute causally to the cognitive side effects of ECT Furthermore, given that cholinergic signalling is influenced by anti-inflammatory actions [70], microglial activation might contribute to ECS-induced cholinergic modifications. However, it is important to note that immune system activation might also be important in the mediation of the therapeutic effects of ECT [71].

It is important to realize that additional mechanisms might be involved in the induction of cognitive deficits by ECT. For example, it has been suggested that ECT sessions result in saturation of long-term potentiation (LTP), a process mediated by activation of glutamatergic NMDA receptors, thereby reducing capacity for further neuroplasticity necessary for memory formation. In addition, ECS is known to be a strong inducer of neurogenesis [12,13]., and it has recently been suggested that excessive neurogenesis might decrease memory retention by interfering with existing synaptic connections [72].

The view that hippocampal LTP and plasticity are involved in the mediation of ECT-induced cognitive dysfunction is supported by rodent studies demonstrating impaired induction of LTP after repeated ECS, combined with evidence that ECS itself increases LTP [73–75]. In addition, LTP could be prevented in rodents by anesthesia with the NMDA receptor antagonist ketamine during ECS sessions [74,76,77]. Human studies confirm a positive effect of ketamine on ECT-induced cognitive side effects [78–80]. It would be highly interesting to study the effects of ketamine anesthesia during ECS sessions on LTP and cognition in the CSSxECS paradigm.

## 5. Limitations

Several limitations should be kept in mind. First, the primary aim of developing a model in which depression-relevant behavioral changes are reversed by ECS could not be achieved This was in part because the combination of CSS and repeated anaesthesia resulted in a loss of the usual CSS effect of increased fear learning-memory, and in part because ECS failed to normalize increased fatigue. This is in contrast to studies in which it has been demonstrated that the CSS-induced increase in fear memory is reversed by repeated administration of escitalopram [33] and the CSS-induced decrease in operant behavior for reward is partly reversed by agomelatine [34]. In addition, as our primary goal was to study reversal of depression-like behavior instead of cognitive deficits, we did not incorporate an extensive test battery for cognitive function. Our evidence for cognitive dysfunction is mainly derived from the fear learning-memory paradigm, in combination with the observation that ECS mice failed to show normal habituation to the treadmill fatigue test, an effect that is likely related to impaired learning. In future studies, tests for other types of memory, such as spatial memory and episodic memory, should also be included. Furthermore, the ECS protocol applied in this study varies on several points from a clinical ECT protocol. For example, isoflurane anesthesia, though common in ECS experiments, is not commonly used for clinical ECT procedures. Instead the use of injection anesthetics such as propofol or methohexital is preferred in a clinical setting. Administration of injection anesthetics in mice however poses practical challenges and may induce stress, which itself could influence subsequent behavioral and immunohistochemical read-out parameters. The same applies to the use of muscle relaxants, which are commonly used in clinical practice but not in animal experiments because of practical challenges posed by the need for ventilation.

In addition, ECT is typically given 2–3 times per week and current dose is titrated to ensure that the minimal dose necessary to achieve a seizure of sufficient intensity is used. In the current study mice underwent ECS on a daily basis for 10 days and fixed stimulus parameters were used. It has been suggested that higher stimulus intensity may be associated with increased severity of cognitive side effects [81]. However, our understanding of how cognitive side effects are influenced by different stimulus parameters and treatment schedules remains limited. Dose-response experiments including different ECS stimulus parameters and treatment schedules in combination with behavioral assessment of cognition would be invaluable for increasing our understanding of the relation between treatment parameters and cognitive side effects.

Despite these limitations this study provides valuable insights into the cognitive side effects of ECS and mechanisms underlying these effects.

## 6. Conclusion

The primary aim of this study was to establish a model of ECS reversal of depression-relevant, stress-induced behavior, thereby allowing for the study of brain changes underlying ECT in human depression. Stress-induced excessive fear learning-memory did not survive the ECS protocol. Stress-induced fatigue was not reversed by the ECS protocol. A secondary, important aim was to investigate whether ECS induces side-effects similar to those commonly reported for ECT in depressed patients. Indeed, the repeated ECS protocol led to marked anterograde impairment in learning and memory and increased fatigue, both in CSS and control mice. These behavioral deficits co-occurred with decreased hippocampal ChAT activity and increased hippocampal microglial activity. These same changes might contribute to the pathophysiology underlying the important side effects that need to be better understood and managed in the application of ECT to patients with treatment-resistant depression.

## Supporting information

S1 Table Body weight. (PDF)

S2 Table Fear conditioning and expression. (PDF)

S3 Table Treadmill fatigue test. (PDF)

S4 Table Hot plate test. (PDF)

S5 Table ARRIVE guidelines checklist. (PDF)
