# Supplementary material for: Mouse repeated electroconvulsive seizure (ECS) does not reverse social stress effects but does induce behavioral and hippocampal changes relevant to electroconvulsive therapy (ECT) side-effects in the treatment of depression
Source: PLoS One. 2017 Sep 14;12(9):e0184603. doi: 10.1371/journal.pone.0184603 (PMC5598988; doi:10.1371/journal.pone.0184603)
Supplement: S1 Table — (PDF) [file pone.0184603.s001.pdf]

S1: Body weight

Body weight block 1-4 (g)

	Control (n=22)		CSS (n=22)	
	Mean	sem	Mean	sem
Block 1	29,19	0,26	29,31	0,48
Block 2	29,83	0,29	29,57	0,38
Block 3	30,44	0,31	30,02	0,45
Block 4	30,69	0,28	30,13	0,42

Δ body weight block 1-4

	Control (n=22)		CSS (n=22)	
	Mean	sem	Mean	sem
Block 1	1,19	0,10	0,93	0,08
Block 2	1,00	0,12	1,73	0,13
Block 3	1,04	0,11	1,25	0,10
Block 4	1,10	0,12	1,54	0,16

Body weight block 4-6 (g)

	Control Sham		Control ECS		CSS sham		CSS ECS	
	Mean	sem	Mean	sem	Mean	sem	Mean	sem
Block 4	30,82	0,38	30,58	0,41	30,00	0,52	30,28	0,69
Block 5	30,73	0,36	29,93	0,32	30,16	0,56	29,98	0,75
Block 6	30,55	0,41	28,97	0,39	30,30	0,50	29,64	0,81
