# Supplementary material for: Mouse repeated electroconvulsive seizure (ECS) does not reverse social stress effects but does induce behavioral and hippocampal changes relevant to electroconvulsive therapy (ECT) side-effects in the treatment of depression
Source: PLoS One. 2017 Sep 14;12(9):e0184603. doi: 10.1371/journal.pone.0184603 (PMC5598988; doi:10.1371/journal.pone.0184603)
Supplement: S2 Table — (PDF) [file pone.0184603.s002.pdf]

S2: Fear conditioning and expression

Fear conditioning: freezing during ITI (% of time)

	Control Sham (n=12)		Control ECS (n=12)		CSS sham (n=11)		CSS ECS (n=11)	
ITI	Mean	sem	Mean	sem	Mean	sem	Mean	sem
1	11,37	4,76	1,43	0,56	21,63	5,90	0,90	0,40
2-3	27,33	4,65	6,90	2,12	30,46	5,96	11,13	4,79
4-5	22,08	3,88	13,81	3,98	24,49	4,67	18,22	6,97

Fear conditioning: freezing during CS (% of time)

	Control Sham (n=12)		Control ECS (n=12)		CSS sham (n=11)		CSS ECS (n=11)	
CS	Mean	sem	Mean	sem	Mean	sem	Mean	sem
1-2	19,73	6,29	16,13	3,74	27,29	8,34	15,99	4,96
3-4	29,16	6,55	12,03	4,47	40,78	7,47	15,51	8,12
5-6	32,51	6,44	21,38	4,98	37,30	5,63	20,63	8,12

Fear expression: freezing during ITI (% of time)

	Control Sham (n=12)		Control ECS (n=12)		CSS sham (n=11)		CSS ECS (n=11)	
ITI	Mean	sem	Mean	sem	Mean	sem	Mean	sem
1	22,50	5,03	8,28	2,69	25,23	5,71	5,45	2,12
2-3	22,15	5,31	12,84	3,50	22,65	5,60	4,91	3,48
4-5	21,88	3,60	7,73	1,97	22,05	7,08	4,25	1,25

Fear expression: freezing during CS (% of time)

	Control Sham (n=12)		Control ECS (n=12)		CSS sham (n=11)		CSS ECS (n=11)	
CS	Mean	sem	Mean	sem	Mean	sem	Mean	sem
1-2	35,58	7,80	10,56	3,27	32,30	6,45	10,10	4,43
3-4	34,60	7,21	8,79	2,76	33,62	7,30	5,45	4,14
5-6	26,84	4,97	10,74	2,35	29,36	8,64	6,65	2,94
