# Supplementary material for: Mouse repeated electroconvulsive seizure (ECS) does not reverse social stress effects but does induce behavioral and hippocampal changes relevant to electroconvulsive therapy (ECT) side-effects in the treatment of depression
Source: PLoS One. 2017 Sep 14;12(9):e0184603. doi: 10.1371/journal.pone.0184603 (PMC5598988; doi:10.1371/journal.pone.0184603)
Supplement: S3 Table — (PDF) [file pone.0184603.s003.pdf]

S3: Treadmill fatigue test

Habituation phase: cumulative duration of shocks (s)

	Sham		ECS	
	**Mean**	**Sem**	**Mean**	**Sem**
**Control**	10,38	0,92	12,72	1,57
**CSS**	10,81	1,62	13,25	2,05

Test phase: cumulative duration of shocks (s)

	Sham		ECS	
	**Mean**	**sem**	**Mean**	**sem**
**Control**	2,35	0,40	5,53	0,98
**CSS**	5,67	1,22	6,76	1,20

Group size

Control x sham: n=12

Control x ECS: n=12

CSS x Sham: n=11

CSS x ECS: n=11
