# Supplementary material for: Mouse repeated electroconvulsive seizure (ECS) does not reverse social stress effects but does induce behavioral and hippocampal changes relevant to electroconvulsive therapy (ECT) side-effects in the treatment of depression
Source: PLoS One. 2017 Sep 14;12(9):e0184603. doi: 10.1371/journal.pone.0184603 (PMC5598988; doi:10.1371/journal.pone.0184603)
Supplement: S4 Table — (PDF) [file pone.0184603.s004.pdf]

S4: hot plate test

Latency to first display pain-related behavior (s)

	**Sham**		**ECS**	
	**Mean**	**Sem**	**Mean**	**Sem**
**Control**	30,83333	3,218868	25,83333	2,151227
**CSS**	36,16667	5,381553	28,83333	5,101743

Group size

Control x sham: n=6

Control x ECS: n=6

CSS x Sham: n=6

CSS x ECS: n=6
